# Wire- and magnetic-seed-guided localization of impalpable breast lesions: iBRA-NET localisation study

**DOI:** 10.1093/bjs/znab443

**Published:** 2022-01-28

**Authors:** Rajiv V. Dave, Emma Barrett, Jenna Morgan, Mihir Chandarana, Suzanne Elgammal, Nicola Barnes, Amtul Sami, Tahir Masudi, Sue Down, Chris Holcombe, Shelley Potter, Santosh K. Somasundaram, Matthew Gardiner, Senthurun Mylvaganam, Anthony Maxwell, James Harvey, iBRA-NET Localisation Study collaborative, A. Tanska, A. Hurley, A. Leusink, E. St John, I. Giono, K. Shanthakunalan, K. Harborough, K. Shenton, N. Gonen, Q. Ain, R. O’Connell, R. Law, V. Teoh, Z. Yan, A. Gaber Eltatawy, T. Rattay, A. Micha, M. Faheem, A. Tenovici, C. Baban, G. Ahmed, M. Joshi, K. Contractor, M. P. Charalambous, M. Kharashgah, M. Hanief, A. Milica, A. Khan, A. Bell, B. Smith, C. Sproson, C. Hollywood, K. A. Hodgkins, C. L. Rutherford, D. Thekkinkattil, D. Shanthakumar, E. Rahman, N. Amulya Mullapudi, A. Morad, E. Quinn, F. Moura, H. Bromley, J. Chen, L. Walter, M. Preston, N. Neyaz, S. Jafferbhoy, R. Osborne, E. Borg, E. Lumley, K. Wijesinghe, F. A. Ross, T. Davies, S. Tovey, H. Fatayer, I. J. Whitehead, J. Mondani, K. James, L. Darragh, T. Kiernan, U. Sridharan, S. Ashford, S. Laws, N. Robson, M. R. A. Matias, R. L. Wilson, S. H. Ali, M. Salman, M. Buhleigah, R. Rathinaezhil, S. Hignett, T. D. Schrire, W. Lambert

**Affiliations:** The Nightingale Breast Cancer Centre, Wythenshawe Hospital, Manchester University NHS Foundation Trust, Manchester, UK; Department of Medical Statistics, Manchester University Hospitals NHS Foundation Trust, Wythenshawe Hospital, Manchester, UK; Department of Oncology and Metabolism, University of Sheffield Medical School, Sheffield, UK; Breast Unit, Lincoln County Hospital, United Lincolnshire Hospitals NHS Trust, Lincoln, UK; Breast Unit, University Hospital Crosshouse, NHS Ayrshire and Arran, Kilmarnock, UK; The Nightingale Breast Cancer Centre, Wythenshawe Hospital, Manchester University NHS Foundation Trust, Manchester, UK; Breast Unit, Lincoln County Hospital, United Lincolnshire Hospitals NHS Trust, Lincoln, UK; Breast screening and assessment unit, Rotherham General Hospital, Rotherham NHS Foundation Trust, Rotherham, UK; Breast Unit, James Paget University Hospital, Great Yarmouth, UK; Breast Unit, Liverpool University Hospitals Foundation Trust, Liverpool, UK; National Institute for Health Research Bristol Biomedical Research Centre, University Hospitals Bristol and Weston NHS Foundation Trust, Bristol, UK; Bristol Breast Care Centre, North Bristol NHS Trust, Bristol, UK; Breast Screening Unit, Royal Lancaster Infirmary, Lancaster, UK; Department of Plastic Surgery, Frimley Health NHS Foundation Trust, Slough, UK; Kennedy Institute of Rheumatology, University of Oxford, Oxford, UK; Health Education West Midlands, Royal Wolverhampton NHS Trust, Wolverhampton, UK; The Nightingale Breast Cancer Centre, Wythenshawe Hospital, Manchester University NHS Foundation Trust, Manchester, UK; Division of Informatics, Imaging & Data Sciences, Faculty of Biology, Medicine and Health, University of Manchester, Manchester, UK; The Nightingale Breast Cancer Centre, Wythenshawe Hospital, Manchester University NHS Foundation Trust, Manchester, UK; Division of Cancer Sciences, Faculty of Biology, Medicine and Health, University of Manchester, Manchester, UK

## Abstract

**Background:**

Wire localization is historically the most common method for guiding excision of non-palpable breast lesions, but there are limitations to the technique. Newer technologies such as magnetic seeds may allow some of these challenges to be overcome. The aim was to compare safety and effectiveness of wire and magnetic seed localization techniques.

**Methods:**

Women undergoing standard wire or magnetic seed localization for non-palpable lesions between August 2018 and August 2020 were recruited prospectively to this IDEAL stage 2a/2b platform cohort study. The primary outcome was effectiveness defined as accurate localization and removal of the index lesion. Secondary endpoints included safety, specimen weight and reoperation rate for positive margins.

**Results:**

Data were accrued from 2300 patients in 35 units; 2116 having unifocal, unilateral breast lesion localization. Identification of the index lesion in magnetic-seed-guided (946 patients) and wire-guided excisions (1170 patients) was 99.8 *versus* 99.1 per cent (*P* = 0.048). There was no difference in overall complication rate. For a subset of patients having a single lumpectomy only for lesions less than 50 mm (1746 patients), there was no difference in median closest margin (2 mm *versus* 2 mm, *P* = 0.342), re-excision rate (12 *versus* 13 per cent, *P* = 0.574) and specimen weight in relation to lesion size (0.15 g/mm^2^  *versus* 0.138 g/mm^2^, *P* = 0.453).

**Conclusion:**

Magnetic seed localization demonstrated similar safety and effectiveness to those of wire localization. This study has established a robust platform for the comparative evaluation of new localization devices.

## Teaser

Magseed^®^ localization has been shown to demonstrate safety and effectiveness not inferior to wire localization. Furthermore, this study has established a robust platform for the comparative evaluation of new breast localization devices.

## Introduction

The expansion of breast screening services worldwide^1^ has resulted in identification of increasing numbers of non-palpable breast lesions that require preoperative localization. Historically, wire-guided lesion localization has been performed in the majority of units^2^. Most breast surgeons and breast radiologists have extensive experience using wires, but this technique has several disadvantages. These include migration of the wire, difficult perioperative wire tip localization resulting in excessive excision of normal breast tissue, and logistical challenges. The latter in particular impacts on surgical scheduling as wires need to be placed on the day of surgery. Despite these difficulties, wire localization remains cheap and effective.

Several alternative methods to wire localization have been developed including radio-occult lesion localization (ROLL)^3^, radioactive seed localization, carbon-track marking^4^ and intraoperative ultrasonography^5^. These are limited by radioactivity regulations, difficult intraoperative lesion detection and need for specialist ultrasound training. Recently, additional novel non-radioactive devices have entered the market that use varying methodologies to guide surgeons to the target lesion. These can be placed in the breast or axilla weeks to months prior to surgery. Devices include Savi Scout^®^^6^ (Cianna Medical Inc., Aliso Viejo, California, USA), LOCalizer™ radiofrequency identification (RFID) tags^7^ (Hologic, Santa Carla, California, USA), Magseed®^8^ (Endomagnetics Inc., Cambridge, UK), Pintuition^®^^9^ (Sirius Medical, Eindhoven, Netherlands) and MOLLI™^10^ (MOLLI surgical, Toronto, Canada). The above devices differ notably in their mode of action, and in the absence of comparative evidence, product selection is largely dependent on surgeon preference.

Magseed^®^ was the first of these newer-generation devices to gain CE marking (2017) following early clinical studies proving early efficacy^11^. Emerging data from users demonstrate that the device can localize lesions accurately, and may reduce re-excision rates, pain^12^ and excision specimen weight^13,14^. These results must be interpreted with caution as the majority of data sets are from single-site case series often reported without a control group and clear prespecified outcome measures. A recent meta-analysis compared wire-guided lesion localization with magnetic seed; however, only one study contained a control group of wire localization and an intervention group of magnetic seed^8,13^ and RFID^7^. Although these findings were supportive of the technique, there remains the need for high-quality research to establish the safety and effectiveness of magnetic seed localization, to determine the key outcomes and how it compares with standard wire localization.

It is likely that even more devices will enter the market in the coming years and both surgeons and patients will require data on efficacy and safety^15^. Although randomized clinical trials are ideal, these are challenging in the context of breast lesion localization. Techniques need to be stable and standardized, and adopted by sufficient number of surgeons. Efficacy data are required so that further studies can be powered adequately. An alternative approach is a well powered multicentre observational study, within an IDEAL (idea, development, exploration, assessment, long-term study) framework^16^ and the use of shared learning to minimize learning curve effects. IDEAL framework phases 2a (development) and 2b (exploration) focus on studies that examine benefits of a device or technique, indications of use and the ability to be adopted by a wider group of surgeons, and to develop feasibility data for a future trial.

The iBRA-NET (implant Breast Reconstruction evaluation-NETwork) Localisation Study is an IDEAL 2a/2b platform study that aims to compare new localization devices with the standard of wire localization. The results from phase 1 of the study, a national practice questionnaire to understand current practice, were reported previously^2^. The results from the first two comparative arms of the IDEAL 2a/2b platform study investigating the outcomes of wire-guided and magnetic-seed-guided localization are reported here.

## Methods

### Study design and participants

The iBRA-NET Localisation Study is a UK-based national, multicentre, prospective, IDEAL stage 2a/2b platform cohort study, with embedded novel shared-learning methodology, that compared safety and effectiveness of magnetic-seed- *versus* wire-guided breast lesion localisation^17^. The shared learning methodology and results will be reported elsewhere.

All UK breast and plastic surgical units performing wire or magnetic seed breast localization were invited to participate in the study, through national professional organizations, namely the Association of Breast Surgery, National Trainee Research Collaborative Network, Mammary Fold Research Network and the iBRA-NET network of surgeons.

Women aged 16 and over, who had breast-conserving surgery requiring a preoperative localization procedure were recruited consecutively to the study between August 2018 and August 2020. Due to potential interference with detection of an implanted magnetic seed, patients were excluded from the magnetic seed arm if they had received iron oxide injection in the previous 6 months or had a pacemaker or implantable electronic device in their chest wall. Ethics approval was not required, as this was a service evaluation, as defined by the Health Research Authority decision tool (http://www.hra-decisiontools.org.uk/research/). Each participating centre was required to obtain local audit approvals and register the study before commencing recruitment—consistent with the methods reported previously for multicentre prospective trainee collaborative studies^18,19^. Patient consent was not required for routine clinical data collection. Study data were collected in an anonymized format and managed using REDCap electronic data capture tools hosted at Kennedy Institute of Rheumatology, University of Oxford^20^. The study involved the collection of clinical outcome data as routinely recommended by UK guidelines for good practice and outcomes were assessed against their quality standards^21,22^.

### Procedures

Study centres recruited consecutive patients undergoing breast localization procedures, with modality of lesion localization (magnetic seed *versus* wire) depending on local availability and policy. Units recruited into either one (if only performing wire localization or having switched to magnetic seed) or both arms of the study depending on local localization practice. Centres offering magnetic seed localization were provided with patient information leaflets and a suggested protocol for magnetic seed localization to ensure consistent quality in insertion, localization and surgical excision (*Appendix S1*), but participating units were able to perform the procedures according to their routine practice. Wire-guided localization was performed on the morning of surgery in all patients, whereas magnetic-seed-guided localization could be performed in advance.

### Quality assurance

In wire-guided localization, it was expected that the operating surgeon should have completed a minimum of 10 wire-guided wide local excisions in the last year. For magnetic seed localization surgery, it was expected that the participating unit should have adopted magnetic seed as their method of localization and were not just trialling the device. This was to ensure that there was adequate expertise in both radiological placement and surgical removal of the magnetic seed. Individual surgeons must have completed a minimum of five successful magnetic seed localization cases and have completed their local training requirements before recruiting to the study.

### Outcomes

The primary outcome was effectiveness, defined as the identification and successful surgical removal or partial removal of the index lesion in the primary operation (or clip/fibrosis in the event of neoadjuvant therapy), based on final pathology. Secondary outcomes were safety (defined as proportion of patients having a peri- and postoperative complication, and complications related to the device (*Appendix S2*)); close margins (defined as ductal carcinoma *in situ* (DCIS)/invasive cancer less than 1 mm from nearest margin); weight of wide local excision specimen (in grams); breast reoperation rate (planned and unplanned); cancellation on day of surgery (proportion of patients cancelled within 24 hours of time of surgery); and the duration and start time of the surgical procedure. In order to account for lesion size in the assessment of excision weight, the authors used size as a dominator over two dimensions, reporting this as weight/size^2^, in g/mm^2^.

### Comparisons of magnetic seed *versus* wire

**Fig 1:**
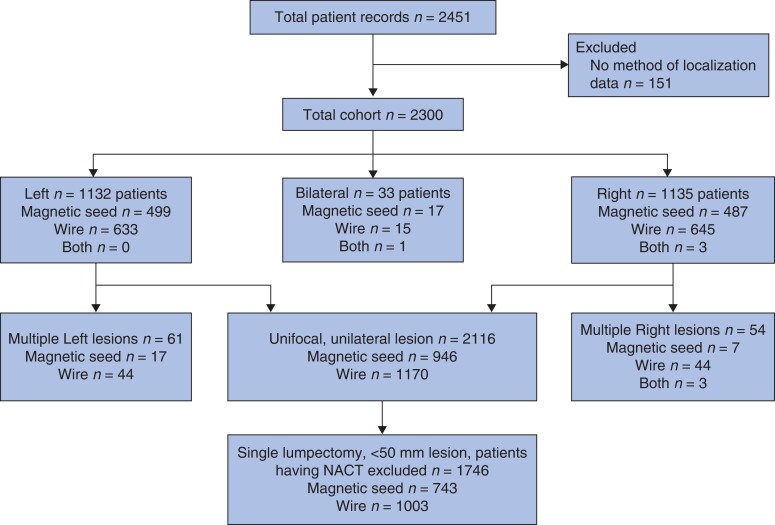
Flow chart of patients included in study NACT, received neoadjuvant chemotherapy

For ease of comparison, only patients having unifocal, unilateral lesion localization were considered. For comparison of surgical parameters, a subset of this group were included, that is patients having a lumpectomy for a single unifocal lesion less than 50 mm in size (cT1 and cT2 only). In this comparison patients operated after neoadjuvant therapy were excluded. If localization was stereotactic-guided, then preoperative size on mammography was used, if localization was ultrasound-guided, then size on ultrasonography was used. Patients having two separate lumpectomies or those having a therapeutic mammoplasty or volume replacement with lipofilling or perforator flap were excluded from this level of analysis (*Fig. 1*).

### Statistical analysis

The failure rate of wires in the literature is 0.6 per cent^23^ giving an identification rate of 99.4 per cent. A clinically significant difference between techniques was considered to be less than 0.9 per cent. Assuming both methods to have an identification rate of 99.4 per cent, the power calculation (upper limit of the observed one-sided 95 per cent confidence interval for the difference between identification rates (magnetic seed *versus* wire) to be less than 0.9 per cent with 80 per cent power) indicated that the sample size should be 1000 patients per group. Simple summary statistics were calculated for each outcome and data were tested for distribution and differences between groups using unpaired *t*-tests, Mann–Whitney U tests and chi-squared tests as appropriate. Analyses were conducted using Stata^®^ IC, version 14 (StataCorp, College Station, Texas, USA).

## Results

There were 2451 patients recruited from 35 UK breast units, of whom 151 were excluded due to incomplete data. The final cohort therefore consisted of 2300 patients (*Fig. 1*). A total of 1003 patients had magnetic-seed-guided localization, 1296 patients had wire-guided localization and four patients had both magnetic-seed- and wire-guided localization. Bilateral lesion localization was required for 33 patients. A total of 120 patients had multifocal/multicentric lesion localization (*Table S1*). Magnetic seed was used infrequently compared with wire for localization of a second lesion, 22.5 *versus* 77.5 per cent of second lesions (*Table S1*). Where three lesions were present, magnetic seed was not used and all three had ultrasound-guided wires placed. There were no patients in the database with bilateral multifocal disease.

### Primary endpoint

Patients within the two comparator groups were well matched, with no differences in clinicopathological variables (*Table 1*). For magnetic-seed-guided excisions, the lesion was present in the excision specimen in 99.8 per cent of cases (905 of 907 patients, unknown 39 patients), and for wire-guided excisions, the lesion was present in the excision specimen in 99.1 per cent of cases (1150 of 1161 patients, unknown 9 patients) (*Table 2*). There was a statistically significant difference between magnetic seed and wire guidance for the primary endpoint of successful index lesion localization (*P* = 0.048, Fisher’s exact test). Of the 13 cases where the index lesion was not removed in the excision specimen, most (9 patients) were reported to have been removed in the core biopsy, with no residual disease in the excision specimen. Other reasons included localization of incorrect lesion (2 patients), lesion present in shave but not main specimen (1 patient), and pathological complete response after neoadjuvant systemic therapy (1 patient).

**Table 1:** Clinicopathological variables in patients with unifocal, unilateral breast lesions

	Magnetic seed (*n* = 946)	Wire (*n* = 1170)	*P* ^‡^
Age (years)*	59.9(10.4)	60.4(10.6)	0.210
**BMI (kg/m^2^ )^†^**	27.9 (24.4–32.1)	28 (24–32)	0.555
Unknown	93	235	
**Lesion size (mm)^†^**	*n* = 764	*n* = 931	
Mammogram	13 (9–20)	13 (9–20)	0.598
Unknown	105	75	
Ultrasound	12 (9–18)	12 (8–17)	0.219
Unknown	182	239	
**Tumour stage**	*n* = 891	*n* = 1147	
Tis	167 (18.7)	256 (22.3)	0.189
T1	541 (60.7)	637 (55.5)	
T2	126 (14.1)	172 (15)	
T3	12 (1.3)	16 (1.4)	
yT0	45 (5.1)	66 (5.8)	
Unknown	55	23	
**Nodal stage**	*n* = 881	*n* = 1147	
N0	747 (84.8)	973 (85.4)	0.543
N1	92 (10.4)	105 (9.2)	
N2	7 (0.8)	11 (1)	
N3	2 (0.2)	8 (0.7)	
yN0	33 (3.7)	42 (3.7)	
Unknown	65	31	
**Histological diagnosis**	*n* = 945	*n* = 1169	
Invasive ductal and lobular	592 (62.6)	715 (61.2)	0.051
DCIS	172 (18.2)	264 (22.6)	
Mixed invasive/DCIS	120 (12.7)	127 (10.9)	
Other	61 (6.5)	63 (5.4)	
Unknown	1	1	
**Neoadjuvant therapy**	*n* = 946	*n* = 1170	
Chemotherapy	100 (10.6)	117 (10)	0.679
Endocrine therapy	34 (3.6)	50 (4.3)	
None	812 (85.8)	1003 (85.7)	
**Surgery**	*n* = 943	*n* = 1170	
Lumpectomy for single lesion	759 (80.5)	1009 (86.2)	0.002
Mammoplasty involving skin/volume reduction	164 (17.4)	142 (12.1)	
Lumpectomy for multiple lesions	4 (0.4)	8 (0.7)	
Other	16 (1.7)	11 (0.9)	
Unknown	3	-	
**Concurrent axillary surgery**	*n* = 935	*n* = 1168	
No	225 (24.1)	328 (28.1)	0.095
Sentinel node biopsy	658 (70.4)	784 (67.1)	
Axillary clearance	52 (5.6)	56 (4.8)	
Unknown	11	2	

Values in parentheses are percentages unless indicated otherwise; *values are mean(s.d.), ^†^values are median (i.q.r.). DCIS, ductal carcinoma *in situ*. ^‡^*t*–test was used for Age, Mann–Whitney *U* test was used for BMI, mammogram, ultrasound, and Chi squared was used for all the others namely Tis, N0, Invasive ductal and lobular, chemotherapy, lumpectomy for single lesion, concurrent axillary surgery (No).

**Table 2:** Primary endpoint: localization success of wire- and magnetic-seed-guided localization

Successful localization	Magnetic seed			Wire			*P*
	Stereo (*n* = 282)	US (*n* = 664)	Total (*n* = 946)	Stereo (*n* = 337)	US (*n* = 833)	Total (*n* = 1170)	
**No**	2 (0.7)	-	2 (0.2)	8 (2.4)	3 (0.4)	11 (0.9)	**0.048**
**Yes**	265 (99.3)	640 (100.0)	905 (99.8)	325 (97.6)	825 (99.6)	1150 (99.1)	
**Unknown**	15	24	39	4	5	9	

Values in parentheses are percentages. Stereo, stereotactic localisation; US, ultrasonographic localisation. Fisher's exact test, comparing "Total Magseed" *versus* "Total Wire". *Fisher's exact test.

### Secondary endpoints

For comparison of surgical parameters, only patients having a unilateral lumpectomy for a single unifocal lesion less than 50 mm were included, and those having neoadjuvant therapy were excluded. When magnetic-seed-guided surgery (743 patients) and wire-guided surgery (1003 patients) were compared, there were no significant differences in closest/involved margin, rates of re-excision, rates of routine shaves taken during surgery, duration of procedure, and specimen weight/size^2^ (*Table 3*).

**Table 3:** Surgical outcomes following lumpectomy for cT1/cT2 unifocal, unilateral lesions guided by magnetic seed and wire. Patients having neoadjuvant chemotherapy were excluded

	Magnetic seed			Wire			*P* ^‡^
	Stereo	USS	Total	Stereo	USS	Total	
	(*n* = 227)	(*n* = 516)	(*n* = 743)	(*n* = 266)	(*n* = 737)	(*n* = 1003)	
**Margins**	*n* = 212	*n* = 478	*n* = 690	*n* = 254	*n* = 720	*n* = 974	
Positive (0 mm at ink)	26 (12.3)	66 (13.8)	92 (13.3)	43 (16.8)	103 (14.3)	146 (15.0)	0.342
Unknown	15	38	53	12	17	29	
Closest margin (mm)*	2 (0–55)	2 (0–20)	2 (0–55)	2 (0–20)	2 (0–55)	2 (0–35)	0.400
**Orientation of closest margin**	*n* = 212	*n* = 477	*n* = 689	*n* = 252	*n* = 707	*n* = 959	
Anterior	44 (20.8)	107 (22.4)	151 (21.9)	53 (21)	192 (27.2)	245 (25.3)	0.137
Posterior	31 (14.6)	102 (21.4)	133 (19.3)	49 (19.4)	146 (20.7)	195 (20.5)	
Radial	137 (64.6)	268 (56.2)	405 (58.8)	150 (59.5)	369 (52.2)	519 (54.2)	
Unknown	15	39	54	14	30	44	
**Re-excision**	*n* = 216	*n* = 491	*n* = 707	*n* = 260	*n* = 730	*n* = 990	
Required	27 (12.5)	60 (12.2)	87 (12.3)	44 (16.9)	87 (11.9)	131 (13.2)	0.574
Unknown	11	25	36	6	7	13	
**Of which, residual disease present**	*n* = 25	*n* = 58	*n* = 83	*n* = 42	*n* = 82	*n* = 124	
Yes	8 (32)	21 (36.2)	29 (34.9)	15 (35.7)	26 (31.7)	41 (33.1)	0.780
Unknown	2	2	4	2	5	7	
**Routine shaves/margins taken during surgery?**	*n* = 227	*n* = 516	*n* = 743	*n* = 266	*n* = 737	*n* = 1003	
No	79 (34.8)	186 (36.0)	265 (35.7)	76 (28.6)	280 (38)	356 (35.5)	0.941
**Orientation of shave/margin taken** ^§^	*n* = 148	*n* = 330	*n* = 478	*n* = 190	*n* = 457	*n* = 647	
Anterior	7 (4.7)	17 (5.2)	24 (5.0)	13 (6.8)	28 (6.1)	42 (6.5)	0.350
Posterior	22 (14.9)	64 (19.4)	86 (18)	39 (20.5)	79 (17.3)	119 (18.2)	0.916
Radial	131 (88.5)	283 (85.8)	414 (86.6)	175 (92.1)	431 (94.3)	606 (93.7)	<0.001
Number of radial shaves*	1 (1–4)	2 (1–4)	2 (1–4)	2 (1–4)	2 (1–4)	2 (1–4)	
**Duration of procedure**	*n* = 213	*n* = 483	*n* = 696	*n*= 261	*n* = 728	*n* = 989	
Duration (min)^†^	60 (45–75)	65 (52–84.5)	60.5 (50–81)	60 (45–75)	60 (45–85)	60 (45–82)	0.100
Unknown	14	33	47	5	9	14	
**Specimen weight/size^2^**	*n*= 186	*n* = 458	*n* = 644	*n* = 227	*n* = 675	*n* = 902	
Specimen (g/mm^2^)^†^	0.159 (0.06–0.42)	0.148 (0.07–0.27)	0.15 (0.07–0.31)	0.133 (0.03–0.94)	0.14 (0.07–0.29)	0.138 (0.06–0.32)	0.453
Unknown	41	58	99	39	62	101	

Values in parentheses are percentages unless indicated otherwise; *values are median (range); ^†^values are median (i.q.r.). ^§^Not mutually exclusive, so for each patient, multiple shaves have been taken. ^‡^Mann–Whitney *U* test for continuous variables and chi-squared test for categorical variables.

A failed localization, where a second method of localization was required (for example magnetic seed or wire not placed in index lesion) was a statistically more common occurrence in wire-guided localization (23 of 1162 patients, 1.98 per cent, unknown 8 patients) compared with magnetic-seed-guided localization (15 of 913 patients, 1.64 per cent, unknown 33 patients) (*P* = 0.032). Magnetic seed was also less likely to be dislodged from the lesion during surgery compared with wire (0.4 *versus* 1.4 per cent, *P* = 0.039, *Table 4*). There were no differences in peri- and postoperative complications related to surgery (*Table 4*). There were eight patients where the magnetic seed was not detectable transcutaneously, but no procedures were abandoned, hence surgeons were able to complete the procedure despite initial difficulty in transcutaneous detection.

**Table 4:** Surgical complications in magnetic-seed- and wire-guided surgery

	Magnetic seed (*n* = 946)	Wire (*n* = 1170)	Total (*n* = 2116)	P*
**Perioperative complications/localization**	*n* = 902	*n* = 1163	*n* = 2065	
None	891 (98.8)	1127 (96.9)	2018 (97.7)	0.132
Magnetic seed/wire dislodged from lesion	4 (0.4)	16 (1.4)	20 (0.9)	0.039
Index lesion/clip not visible on specimen Xrays	4 (0.4)	9 (0.8)	13 (0.6)	0.406
Procedure abandoned, further localization required	-	-	-	-
Other	3 (0.3)	11 (0.9)	14 (0.7)	0.106
Unknown	44	7	51	
**Postoperative complications/surgery**	*n* = 946	*n* = 1170	*n* = 2116	
Seroma requiring aspiration	14 (1.5)	25 (2.1)	39 (1.8)	0.264
Haematoma requiring aspiration in clinic	3 (0.3)	8 (0.7)	11 (0.5)	0.364
Haematoma requiring surgical evacuation	3 (0.3)	4 (0.3)	7 (0.3)	1.000
Minor wound infection (oral antibiotics)	14 (1.5)	27 (2.3)	41 (1.9)	0.170
Major wound infection (IV antibiotics)	3 (0.3)	7 (0.6)	10 (0.5)	0.527
Major wound infection (drainage/debridement)	1 (0.1)	4 (0.3)	5 (0.2)	0.388
In-hospital complication including systemic complications such as DVT/PE/MI	6 (0.7)	4 (0.3)	10 (0.5)	0.349
Unexpected readmission to hospital within 30 days^†^	7 (0.8)	11 (1)	18 (0.9)	0.676
**Readmission directly related to the localization procedure**				
Reoperation within 30 days^‡^	29 (3.2)	53 (4.6)	82 (4)	0.119
Complications directly related to localization device (magnetic seed or wire)^§^	2 (0.2)	2 (0.2)	4 (0.2)	1.000

Values in parentheses are percentages. *All were done using Chi squared test. ^†^Unknown data in 50 magnetic seed and 18 wire. ^‡^Unknown data in 51 magnetic seed and 19 wire. ^§^Unknown data in 49 magnetic seed and 19 wire. IV, intravenous; DVT, deep venous thrombosis; PE, pulmonary embolism; MI, myocardial infarction.

### Multifocal lesion localization using bracketing

Bracketing, the use of two or more localization devices to define the extent of the lesion(s) that require excision, was employed in 49 patients. There were 12 patients who had bracketing with magnetic seed and 37 with wires. Localization was successful in 100 per cent of the index lesions and 97.7 per cent of the second lesion (43 of 44 patients, data missing on 5 patients). When bracketing was used, the median distance between lesions was 40 (i.q.r. 28–49) mm for magnetic seed and 34 (i.q.r. 26–45) mm for wires.

### Logistical considerations

There was a low rate of cancellation on the day of surgery for both modalities; 0.9 per cent (9 of 997 patients) for magnetic seed, and 0.5 per cent (6 of 1290 patients) for wire and none for dual modality (wire and magnetic seed). No cancellations were related to the localization procedure.

Magnetic seed lesion localization was performed a median of 6 days before surgery (range 0–167 days, i.q.r. 3–12 days). Most surgery was done as a day-case, with a median length of stay being 0 (range 0–11) days for magnetic-seed-guided surgery, and 0 (range 0–7) days for wire-guided surgery. Magnetic-seed-guided surgeries were started earlier in the day (*Fig. S1*).

## Discussion

This study reports on the first arm of the iBRA-NET Localisation Study, and is the largest study to date comparing wire-guided and magnetic-seed-guided localization in breast surgery. Results show that magnetic seed compared equivalently with wire in terms of localization of non-palpable breast lesions. Additionally, reported complications were low demonstrating safety of this innovative localization technique. This study has contributed substantially to the collective safety data of this novel technique, and aims to set a standard for a robust IDEAL 2a/2b evaluation for future surgical devices.

Magnetic seeds have been subject to multiple published evaluations, albeit largely in single-centre, small cohorts. The results of the present study compare to some but are in contrast to others. Early US data were reassuring for accuracy of placement, with 100 per cent (73 patients) successful placement, defined as positioning within 10 mm of the target, most of which (51 of 73 patients, 70 per cent) were located within 1 mm (either directly contacting the target or immediately adjacent to it)^24^. Data from the UK were limited to small single-institution cohorts, with contrasting results. A series from London (128 patients) reported smaller specimen weights, but similar rates of positive margins^14^. A series from Lincoln (137 patients) reported a mean specimen weight of 75.5 g (0.327 g/mm^2^ for comparison) and a re-excision rate of 14.8 per cent. A series from Manchester reported no significant differences in re-excision rate (104 patients, 16 per cent with magnetic seed and 14 per cent wire, *P* = 0.692)^8^. A systemic review and pooled analysis of 1559 procedures from 16 studies concluded that magnetic seed provided an effective, non-inferior alternative to wires, with a successful localization rate of 99.86 per cent (16 studies), and a re-excision rate of 11.19 per cent (12 studies). A re-excision rate was determined based on results from only four studies, comparing a total of 319 magnetic-seed-localized excisions against 507 wires, with a non-significant re-excision rate of 18.5 per cent for magnetic seed and 16.17 per cent for wires^13^.

A series from Shrewsbury (106 patients with magnetic seed *versus* 90 with wire) concluded that there was a significant reduction in re-excision rate from 22.4 to 12 per cent, and average specimen weight from 40 to 27 g^25^. A randomized trial of radioactive seed *versus* wire from Australia demonstrated an improved re-excision rate of 13.9 per cent for radioactive seed (327 patients) *versus* 18.9 per cent for wires (332 patients) (*P* = 0.019)^26^. For the latter study, it must be noted that for the purposes of power calculation, the re-excision rate was expected to be 30 per cent, resulting in an underpowered study. In comparison, margin positivity is reported in the world literature to be between 16.4 and 20 per cent^27,28^. These two studies have demonstrated a much higher rate of re-excision in patients undergoing wire-guided localization, resulting in a potentially false ‘improvement’. The results of the iBRA-NET Localisation Study are in keeping with recent published data from the UK, reflecting more current practice^29^. In a follow-up cost-effectiveness analysis of the use of radioactive seed localization in preventing future re-excisions, data from the ROLLIS study group showed that using seeds is cost-effective. It was concluded that the marginal additional cost when compared with wire localization is less than the cost of reoperations avoided^30^.

The present study demonstrated that magnetic seed localization did not lead to improvement in the majority of secondary surgical outcomes, including re-excision. Wires and magnetic seeds are both highly effective at localizing lesions, and the majority of complications are related to adjuncts to surgery rather than the localization technique. Several single-unit, smaller studies have demonstrated a reduction in re-excision rate after adopting the new technology, particularly for radioactive seeds^31^. However, these studies have selection bias and are generally single site. A randomized trial of radioactive seed localizations from Denmark in 2017^32^ concluded that there were major logistical advantages, but with no differences in positive resection margins (11.8 *versus* 13.3 per cent), duration of the procedure or specimen weight. It is thus plausible that some surgical outcomes are not dependent on the localization device, but rather a reflection of the disease. Re-excision is most commonly required for DCIS at the margins^33^ and is a consequence occult disease being present rather than poor localization. Re-excision is thus most commonly a mismatch between preoperative clinical expectation and postoperative histology.

The limitations of this study are recognized. There were no data collected on distance of magnetic seed placement from the index tumour or on transcutaneous signal detection. The study was not powered for more complex scenarios of non-palpable breast lesion localization, such as multiple lesions and ‘bracketing’. Being an observational study, there was potential for selection bias because localization modality was dependent on surgeon preference and service convenience. Moreover, not all participating surgeons or services offered both localization modalities. The lack of difference in secondary outcomes could be explained by subtleties that are difficult to capture. For example, during localization of the index lesion, on rare occasions, a second lesion is identified. If this were to be found in advance (as is the case with magnetic seed) this would give time for diagnostic biopsy, and surgery could proceed as planned, whereas with wires the operation would have to be postponed.

Magnetic-seed-guided surgery is a novel technique, and most present data were collected during the ‘early years’ of introduction in the UK. Despite all users having completed their initial learning curve, it is feasible that an on-going learning curve may still have a part to play in the lack of expected superiority in re-excision rates and localization success. Additionally, there may be a lack of standardization of practice amongst individual units performing this technique. It was not the intention of the authors to dictate practice in individual units, and it is likely that this evolved during the implementation of the technology, which cannot be controlled. This, however, could be argued to be beneficial in capturing real-world evidence of current practice in varied healthcare settings. The embedded shared learning allowed for dissemination of surgical technique and challenges, and the technique evolving with experience.

This study does not examine clinician and patient satisfaction, or cost analysis which would help to determine the localization modality that individual breast units choose to adopt. Previous studies have reported favourable clinician satisfaction, but no difference in patient satisfaction, although they did report lower preoperative patient anxiety with magnetic seed, when compared with wire^13,14^. This study did not report on the utilization of magnetic seed in axillary surgery, and it has been reported previously that magnetic seed localization appears to be a safe, non-radioactive method to localize axillary lymph nodes accurately before surgery^34^.

The development of magnetic seed and other non-palpable breast lesion localization devices may provide a safe alternative to wires, with logistical benefits. Progressing from the IDEAL 2a/2b design of this present study could involve a randomized study of several localization modalities. This would, however, have to be highly powered, and may not be feasible due to the large range of localization devices available, the difficulties in training and operational standardization of these devices in a study involving multiple surgeons, and the lack of large-scale baseline data for the newer emerging devices. Subsequent arms of the iBRA-NET Localisation Study will, however, report on other localization devices including LOCalizer™ and Savi-Scout^®^. The study design will enable direct comparison with all other arms of the study giving patients and clinicians robust and reliable data to inform them of the effectiveness and operative outcomes. Further work should also include examining patient and clinician preference using a qualitative approach, a cost analysis to evaluate the technology fully, and assessment of the ability to localize lymph nodes in the axilla accurately to facilitate targeted axillary dissection.

## Supplementary Material

znab443_Supplementary_Data

## Collaborators

Local investigators and members of the iBRA-NET Research Collaborative are recognized as PubMed citable collaborators:

The Royal Marsden Hospital, London: A. Tanska, A. Hurley, A. Leusink, E. St John, I. Giono, K. Shanthakunalan, K. Harborough, K. Shenton, N. Gonen, Q. Ain, R. O’Connell, R. Law, V. Teoh, Z. Yan; Glenfield Hospital, Leicester: A. Gaber Eltatawy; University Hospitals of Leicester NHS Trust, Leicester: T. Rattay; St Bartholomew Hospital, London: A. Micha, M. Faheem; The Parapet, King Edward VII Hospital, Frimley Health NHS Foundation Trust, London: A. Tenovici, C. Baban, G. Ahmed, M. Joshi, K. Contractor, M. P. Charalambous, M. Kharashgah, M. Hanief; Basingstoke and North Hampshire Hospital, Basingstoke: A. Milica; Ninewells Hospital, Dundee: A. Khan; University Hospital North Tees, Stockton-on-Tees: A. Bell; Royal Berkshire Hospital, Reading: B. Smith; Rotherham NHS Foundation Trust, Rotherham: C. Sproson; Chesterfield Royal Hospital, Chesterfield: C. Hollywood, K. A. Hodgkins. Gartnavel General Hospital, Glasgow: C. L. Rutherford; Lincoln County Hospital, Lincoln: D. Thekkinkattil; Whipps Cross University Hospital, London: D. Shanthakumar; The Royal Wolverhampton NHS Trust, Wolverhampton: E. Rahman, N. Amulya Mullapudi; Manchester University NHS Foundation Trust, Manchester: A. Morad, E. Quinn, F. Moura, H. Bromley, J. Chen, L. Walter, M. Preston, N. Neyaz, S. Jafferbhoy, R. Osborne; St James University Hospital, Leeds: E. Borg, E. Lumley, K. Wijesinghe; University Hospital Crosshouse, Kilmarnock: F. A. Ross, T. Davies, S. Tovey; North Manchester General Hospital, Manchester: H. Fatayer; Countess of Chester Hospital, Chester: I. J. Whitehead; Nottingham Breast Institute, Nottingham: J. Mondani; Wirral University Teaching Hospital NHS Foundation trust, Birkenhead: K. James; Ulster Hospital, South Eastern Trust, Belfast: L. Darragh; St Helens and Knowsley Teaching Hospitals NHS Trust, Prescot: T. Kiernan, U. Sridharan, S. Ashford; Hampshire Hospitals Foundation Trust, Winchester: S. Laws, N. Robson; Blackpool Victoria Hospital, Blackpool: M. R. A. Matias, R. L. Wilson; Royal Lancaster Infirmary, Lancaster: S. H. Ali; Nobles Hospital, Douglas, Isle of Man: M. Salman; Brighton and Sussex University Hospitals NHS Trust, Brighton: M. Buhleigah, R. Rathinaezhil; Mid Cheshire Hospitals, Crewe: S. Hignett; Southmead Hospital, Bristol: T. D. Schrire; Kingston Hospital NHS Foundation Trust, Kingston-upon-Thames: W. Lambert.
