# Supplementary material for: Wire- and magnetic-seed-guided localization of impalpable breast lesions: iBRA-NET localisation study
Source: Br J Surg. 2022 Jan 28;109(3):274–82. doi: 10.1093/bjs/znab443 (PMC10364683; doi:10.1093/bjs/znab443)
Supplement: znab443_Supplementary_Data [file znab443_supplementary_data.zip › Supplementary_material.docx]

**Supplementary material**

| Lesion 1 | Lesion 2 | n |
| --- | --- | --- |
| Stereo-Magseed (n=9) | Stereo-Magseed | 8 |
|  | USS-Magseed | - |
|  | Stereo-Wire | - |
|  | USS-wire | 1 |
| USS-Magseed (n=16) | Stereo-Magseed | - |
|  | USS-Magseed | 16 |
|  | Stereo-Wire | - |
|  | USS-wire | - |
| Stereo-Wire (n=30) | Stereo-Magseed | - |
|  | USS-Magseed | 2 |
|  | Stereo-Wire | 28 |
|  | USS-wire | - |
| USS-wire (n=65) | Stereo-Magseed | - |
|  | USS-Magseed | 1 |
|  | Stereo-Wire | 7 |
|  | USS-wire | 57 |

**Table S1. Modality of localisation in patients with multifocal disease.**

**Appendix S2**

The following definitions of complications were used:

**1. Peri-operative complications**

**Cancellation of surgery –** patient has breast localisation operation (wide local excision or localised diagnostic excision biopsy) cancelled in the 24 hours prior to the time of the scheduled operation.

**Failed localisation**

A failed localisation where a second method of localisation was required (e.g. Magseed or wire not placed in index lesion). This could be due to: localisation of a lesion that was not the index lesion; localisation missed the index lesion or migration of the localisation device.

**2. Post-operative complications**

**Haematoma** - A collection of blood in the breast

- **Minor –** managed conservatively, or by aspiration in clinic or
- **Major –** requiring surgical evacuation.

**Infection** - A hot, red swollen breast associated with one of the following; a temperature, pus at the wound site, a raised white cell count; a positive wound culture within the first 3 months following surgery. This will be further classified as:

- **Minor** – requiring oral antibiotics only;
- **Major 1** – requiring admission for IV antibiotics and/or debridement;
- **Major 2** – requiring surgical drainage/debridement

**Wound dehiscence –** separation of the skin edges at the wound site.

- **Minor** – treated conservatively;
- **Major** – requiring return to theatre for re-suturing under GA

**In hospital complication –** any complication that occurs during the patient’s initial hospital stay at the time of their surgery. This includes systematic complications such as DVT/PE and procedure specific complications such as haematoma.

**Readmission to hospital –** any re-admission to hospital in the 30 days following surgery directly related to the procedure but excluding re-excision of margins (e.g with infection requiring antibiotics or systemic complications including pulmonary embolus)

**Return to theatre –** Return to the operating theatre at any time during the first 30 days to deal with any complications directly related to the breast surgery.

**Major complication -** Any complication requiring readmission to hospital or return to theatre

**Minor complication** - Any other complication
