# Supplementary figures and images for: Wire- and magnetic-seed-guided localization of impalpable breast lesions: iBRA-NET localisation study
Source: Br J Surg. 2022 Jan 28;109(3):274–82. doi: 10.1093/bjs/znab443 (PMC10364683; doi:10.1093/bjs/znab443)

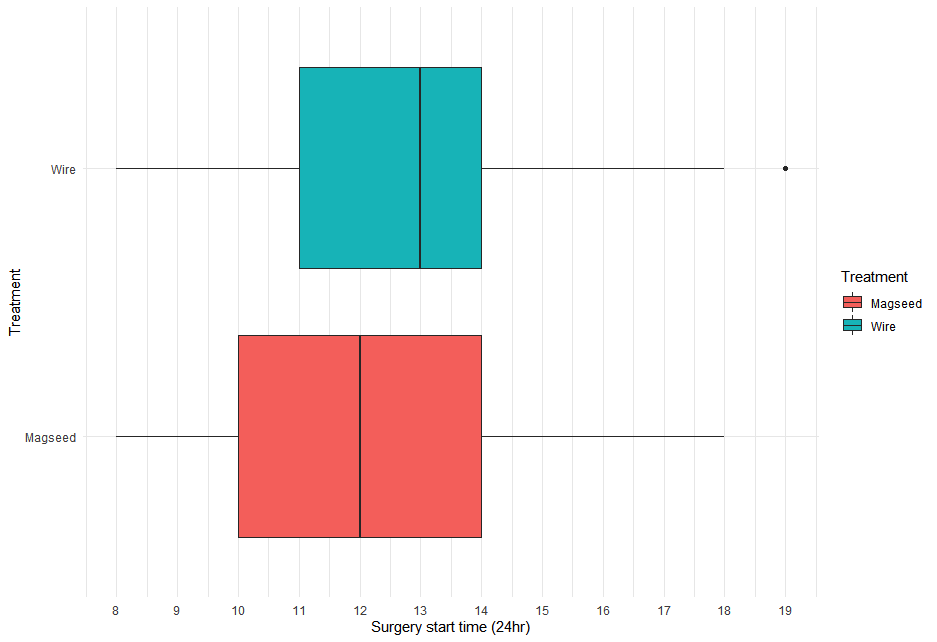

Supplement: znab443_Supplementary_Data [file znab443_supplementary_data.zip › Supplementary_Figure_1.tiff]
